# Rectified Oil of Amber as a Remedy for Hœmorrhoids

**Published:** 1866-08

**Authors:** 


### *Rectified Oil of Amber as a Remedy for Hæmorrhoids.*

The editor of the American Journal of Pharmacy, Mr. William Proctor, Jr., contributes the following interesting communication to the journal of which he is editor:

"Of the large number of persons who suffer from this annoying complaint, very many never consult a physician, and many others after renewed treatment give up the idea of becoming cured, viewing the affliction as some do old ulcers, as a burden to be borne while life continues. Various external applications are constantly prescribed, as an ointment of acetate of lead, tannin or nutgall, and opium, which is often successful in affording relief. Numerous secret nostrums have, from time to time, attracted attention, indicating the prevalence of the disease. Several years ago my curiosity was excited by the repeated calls for rectified oil of amber by a person who was not in any way connected with medicine, and he was asked the use to which it was applied. He said it was for piles, and that he rarely knew it to fail; the numerous calls that had been made being for friends and acquaintances who were sufferers from the complaint. After that, on several occasions where opportunity offered, it was suggested and tried with success, in many cases of piles where the tumors were external and annoying. The manner of its curative action I am not aware of. The oil is applied as a lotion to the tumors, and around the anus where the swellings exist. It occasions a smarting sensation at first, but after several applications the sensitiveness disappears, and the tumors are dissipated. So far as is known to the writer, the influence is entirely local, and does not extend beyond the parts to which it is applied. I am not aware that it has been applied beyond the sphincter ani to the internal tumors, but know of a case wherein both internal and external piles existed, the latter disappearing, and the others continuing to give annoyance. The object of this note is to ask the attention of medical men to the subject, that the actual value of the oil of amber as a remedy for piles may be satisfactorily tested. It may be that in some cases admixture with lard or cerate would be preferable, and in the form of an emulsion, or associated with glycerin or olive oil, it might be applied in the rectum by injection or by a bougie. These are mere suggestions to the physician.

"It is to be regretted that so little genuine oil of amber is to be obtained, as has been conclusively shown by Mr. Ebert, of Chicago (see page 146 of this volume), who finds that it costs as much per ounce to make the oil as it sells for in commerce per pound. Failures may be attributed to the spurious oil made from turpentine and coal oil, shaken with oil of tar and oil of amber."

